# Dysfunctional immunoregulation in human liver allograft rejection associated with compromised galectin-1/CD7 pathway function

**DOI:** 10.1038/s41419-017-0220-3

**Published:** 2018-02-20

**Authors:** Sidong Wei, Ding Cao, Zuojin Liu, Jinheng Li, Hao Wu, Jianping Gong, Yiming Liu, Yakun Wu

**Affiliations:** 1 Department of Hepatobiliary Surgery, People’s Hospital of Zhengzhou, Zhengzhou, 450003 China; 2 Department of Hepatobiliary Surgery, Second Affiliated Hospital, Chongqing Medical University, Chongqing, 400010 China

## Abstract

Regulatory T cells in rejected allograft patients display an inability to control responder T cells. Galectin-1 (Gal1) inhibits responder T cells through binding CD7. We investigated whether the dysfunctional immunoregulation in liver allograft rejection patients results from reduced regulatory T-cell Gal1 expression and/or responder T-cell CD7 expression. Circulating regulatory T cells and responder T cells were profiled from 31 acute rejection transplant patients, 85 transplant patients in remission, and 40 healthy controls. CD7+ and CD7− responder T cells were co-cultured with regulatory T cells to assess regulatory T-cell suppressor function. Gal1-small interfering RNA was used to silence regulatory T-cell Gal1. The CD7+ cell percentage was inversely correlated with AST, ALT, and GGT levels. The proportions of CD7+ responder T cells and Gal1+ regulatory T cells were higher in healthy controls than in transplant patients in remission and lowest in acute rejection transplant patients. Notably, CD7+ responder T-cell susceptibility to Gal1+ regulatory T-cell control was ranked in the same manner. Silencing Gal1 expression in regulatory T cells reduced their ability to suppress CD7+ (but not CD7−) responder T cells. Additionally, the proportions of CD43+ and CD45+ responder T cells were higher in healthy controls than in acute rejection transplant patients. CD43 co-expression (but not CD45 co-expression) on CD7+ responder T cells promoted their apoptosis in a Gal1-dependent manner. In sum, dysfunctional immunoregulation in liver allograft rejection patients can be partly attributed to reduced regulatory T-cell Gal1 expression and reduced responder T-cell CD7 expression. Responder T-cell CD43 downregulation in acute rejection patients may further contribute to reduced responder T-cell responsiveness to regulatory T-cell control.

## Introduction

Allograft rejection remains a critical challenge following liver transplantation, with ~10–20% of adult liver transplant recipients experiencing an acute rejection event within 1 year post transplant^1^. Allograft rejection is characterized by an alloimmune response in which the recipient’s antigen-presenting cells present processed allopeptides to CD4+ T cells^1^. Although long-term survival following transplantation has improved since the early 80s, transplant recipients must continue to take immunosuppressive medications in order to control CD4+ T-cell alloreactivity^2,3^. Unfortunately, immunosuppressive agents raise the transplant recipient’s susceptibility to malignancy, infectious disease, and adverse cardiovascular effects^2,4^. On this basis, improving our understanding of the role of CD4+ T cells in allograft rejection is critical to developing safer and more efficacious strategies for inducing allograft tolerance in transplant recipients.

With regard to this issue, the magnitude of the alloreactive CD4+ T-cell response has been positively linked with the inhibition of thymus-derived CD4+CD25+ T cells (regulatory T cells, T_regs_), a T-cell subset that plays an important role in maintaining immunotolerance^5^. T_regs_ have been shown to induce and maintain allograft tolerance in transplant recipients, while T_regs_ in patients with rejected allografts display an inability to control responder CD4+ T cells^5^. With respect to promoting T_reg_ activity, the lectin galectin-1 (Gal1) has been shown to ameliorate inflammation in animal models of autoimmunity by sparing T_regs_ and Th2 cells while promoting apoptosis in Th1, Th17, and Tc1 cells^6^. These previous findings reveal that Gal1 may play an important role in promoting tolerance in autoimmune disease.

However, the role of Gal1 (if any) in allograft tolerance remains poorly understood, yet there are some promising lines of evidence. For example, the expression of recombinant Gal1 in mice suppresses graft-vs.-host disease, promotes host survival, and prolongs allograft survival^6^. Moreover, administrating recombinant Gal1 to murine recipients of Flt3L-pretreated livers significantly delays allograft rejection through promoting alloreactive T-cell apoptosis and suppressing Th1 and Th17 activity^7^. These findings coincide with those of Garcia et al.^8^, who found that Gal1 levels were significantly higher in stable liver transplant recipients relative to acutely rejecting recipients as well as healthy controls. These combined findings suggest that Gal1 may play an immunosuppressive role in liver transplant recipients.

Although the foregoing research suggests that Gal1 can ameliorate liver allograft rejection by inducing apoptosis of alloreactive T cells and inhibiting Th1 and Th17 responses^6,7^, whether Gal1 acts through ameliorating the underlying T_reg_ defect or bolstering the lowered responsiveness of CD4+ responder T cells to T_reg_ control remains unclear. Therefore, the aim of this study will be to explore the role of Gal1 in liver allograft rejection and particularly to determine whether Gal1 acts by ameliorating defective T_regs_ function, bolstering lowered responsiveness of CD4+ responder T cells to T_reg_ control, or both.

## Results

### Demographic and clinical characteristics of the recruited patients

A total of 156 participants were finally included in this study, consisting of 31 acute rejection transplant patients, 85 transplant patients in remission, and 40 healthy controls. There were no significant differences in age between the three groups (*p* > 0.05, Table 1), while there was a significantly higher proportion of males in the acute rejection group relative to the other two groups (*p* < 0.05, Table 1). The two transplant patients groups contained significantly higher percentages of participants with hepatocellular carcinoma tumors, hepatorenal syndrome, stage 3 encephalopathy, and gastrointestinal bleeds relative to the healthy control group (all *p* < 0.05, Table 1). Moreover, the two transplant patients groups displayed significantly higher levels of total bilirubin and direct bilirubin as well as significantly lower levels of hemoglobin relative to the healthy control group (all *p* < 0.05, Table 1). In addition, the acute rejection group displayed significantly higher levels of aspartate transaminase (AST), alanine transaminase (ALT), total bilirubin, direct bilirubin, gamma-glutamyl transferase (GGT), and white blood cell (WBC) relative to the remission group (all *p* < 0.05, Table 1).

**Table 1:** Demographic and clinical characteristics of study participants

Characteristic	Acute rejection	Remission	Healthy controls
Number (*n*)	31	85	40
Age (range)	46.5 (22.2–66.5)	45.9 (21.3–64.0)	46.3 (21.9–63.5)
Sex (male, %)	77%*	64%	50%
Percentage with HCC tumor(s) (%)	19%*	19%*	0%
Percentage with hepatorenal syndrome (Cr > 3 mg/dl, %)	10%*	8%*	0%
Percentage with stage 3 encephalopathy (%)	35%*	32%*	0%
Percentage with GI bleeds (%)	29%*	25%*	0%
AST (SD)	132.88 (147.95)*^†^	30.20 (13.09)	23.39 (5.05)
ALT (SD)	158.63 (149.30)*^†^	23.75 (18.04)	22.21 (8.42)
Total bilirubin (SD)	2.59 (2.18)*^†^	0.81 (0.29)	0.80 (0.25)
Direct bilirubin (SD)	1.71 (1.76)*^†^	0.35 (0.18)*	0.20 (0.05)
GGT (SD)	177.83 (87.87)*^†^	37.32 (21.87)*	18.94 (3.02)
Albumin (SD)	3.58 (0.47)	3.66 (0.52)	4.36 (0.30)
PT-INR (SD)	1.09 (0.19)	1.01 (0.11)	1.22 (0.05)
Hemoglobin (SD)	8.20 (1.07)*	8.63 (1.71)*	15.08 (1.00)
WBC (SD)	8.05 (5.00)*^†^	5.70 (2.02)	5.83 (1.24)

*p < 0.05 vs. healthy control group, ^†^*p* < 0.05 vs. remission group

### Analysis of responder T cells

**Fig. 1:**
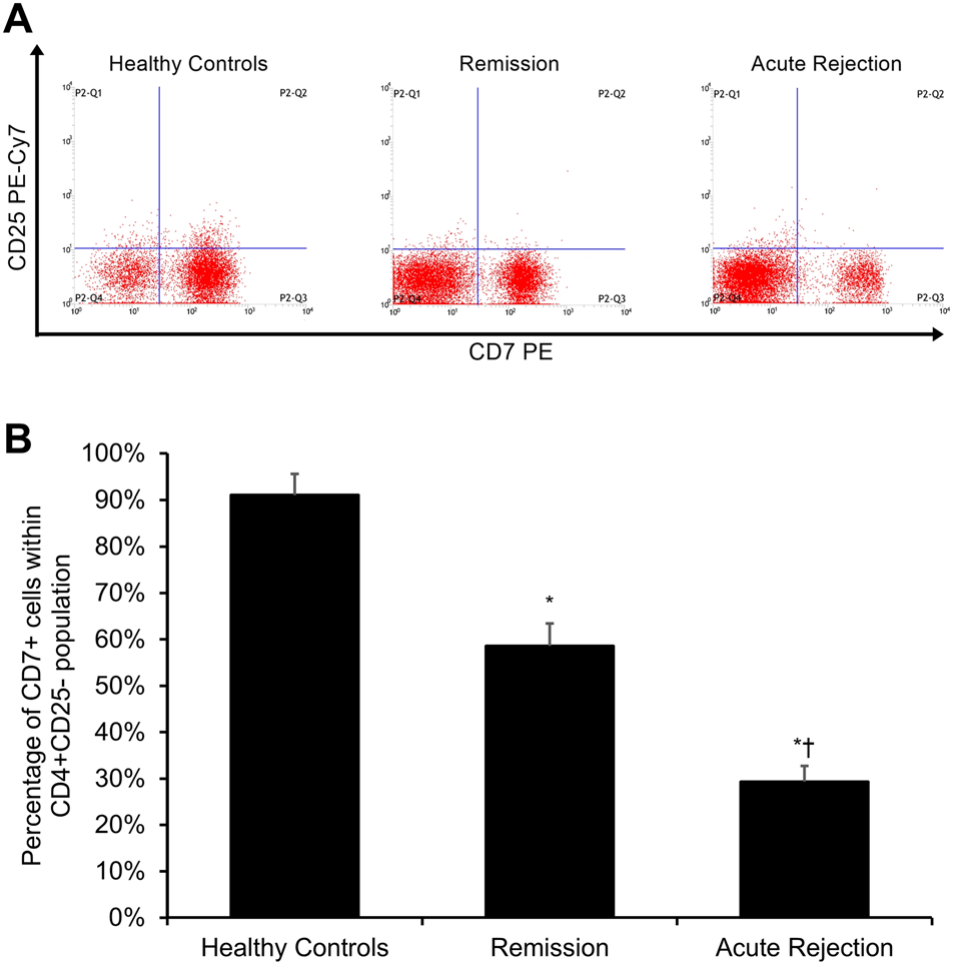
Percentage of CD7+ cells within the CD4+CD25− responder T-cell population. **a** Representative dot plots of CD7 PE (*x*-axis) vs. CD25 PE-Cy7 (*y*-axis) from a healthy control participant (left panel), a transplant patient in remission (middle panel), and an acute rejection transplant patient (right panel). Cells were gated on CD4+ T cells. **b** Percentage of CD7+CD25− cells in healthy controls (*n* = 40), transplant patients in remission (*n* = 85), and acute rejection transplant patients (*n* = 31). Each experiment was performed in triplicate. Results are reported as means ± standard errors of mean (SEMs). *p < 0.05 vs. healthy controls group, ^†^*p* < 0.05 vs. remission group

The proportion of CD4+CD25−CD7+ responder T cells within the overall CD4+CD25− responder T-cell population was significantly lower in transplant patients relative to healthy control subjects (*p* < 0.05, Fig. 1), with the CD4+CD25−CD7+ responder T-cell percentage being significantly lower in acute rejection transplant patients as compared to transplant patients in remission (*p* < 0.05, Fig. 1).

The proportions of CD7+RORC+, CD7+IFNγ+, and CD7+IL-17+ as well as CD7−IFNγ+ and CD7−IL-17+ responder T cells were significantly higher in acute rejection transplant patients relative to healthy control subjects (all *p* < 0.05, Table 2). Moreover, the proportion of CD7−FOXP3+ responder T cells were significantly lower in acute rejection transplant patients as compared to transplant patients in remission and healthy control subjects (*p* < 0.05, Table 2). Relative to CD4+CD25−CD7− responder T cells, CD4+CD25−CD7+ responder T cells from all three patient cohorts contained a significantly higher proportion of T-bet+ cells and a significantly lower proportion of GATA-3+ cells (both *p* < 0.05, Table 2).

**Table 2:** Cell subtype percentages within the CD4+CD25−CD7+ and CD4+CD25−CD7− cell populations

	CD7+			CD7−		
	Acute rejection (%)	Remission (%)	Healthy controls (%)	Acute rejection (%)	Remission (%)	Healthy controls (%)
T-bet+	65.8 ± 0.6^‡^	66.8 ± 0.6^§^	68.0 ± 0.4^¶^	18.5 ± 1.6^‡^	15.1 ± 1.3^§^	11.4 ± 1.7^¶^
GATA-3+	0.5 ± 0.2^‡^	0.5 ± 0.2^§^	0.6 ± 0.2^¶^	2.0 ± 0.5^‡^	3.5 ± 0.9^§^	5.3 ± 0.8^¶^
RORC+	4.1 ± 0.5*	3.1 ± 0.4	2.1 ± 0.4	10.7 ± 0.7	8.7 ± 0.6	6.6 ± 0.9
FOXP3+	2.1 ± 0.6^‡^	2.1 ± 0.6	2.0 ± 0.4	0.3 ± 0.1*^†‡^	1.6 ± 0.6	3.0 ± 1.5
IFNγ+	6.0 ± 0.4*	4.6 ± 0.3	3.1 ± 0.4	8.7 ± 1.0*	6.7 ± 0.8	4.5 ± 0.6
IL-17+	5.4 ± 0.8*	4.3 ± 0.7	3.1 ± 0.6	5.1 ± 1.1*	4.0 ± 0.9	2.8 ± 0.4
IL-10+	2.8 ± 0.4	4.9 ± 0.7	7.2 ± 0.9	6.4 ± 0.9	9.3 ± 1.3	12.6 ± 1.9
TGFβ+	2.3 ± 0.1	4.6 ± 0.3	7.2 ± 1.4	6.0 ± 1.8	7.3 ± 2.3	8.8 ± 0.8

Data reported as means ± SDs

Within the CD7+ subset or CD7− subset: *p < 0.05 vs. healthy control group, ^†^*p *< 0.05 vs. remission group

Within the acute rejection group: ^‡^*p* < 0.05, CD7+ subset vs. CD7− subset

Within the remission group: ^§^*p* < 0.05, CD7+ subset vs. CD7− subset

Within the healthy control group: ^¶^*p* < 0.05, CD7+ subset vs. CD7− subset

### Analysis of T_regs_

**Fig. 2:**
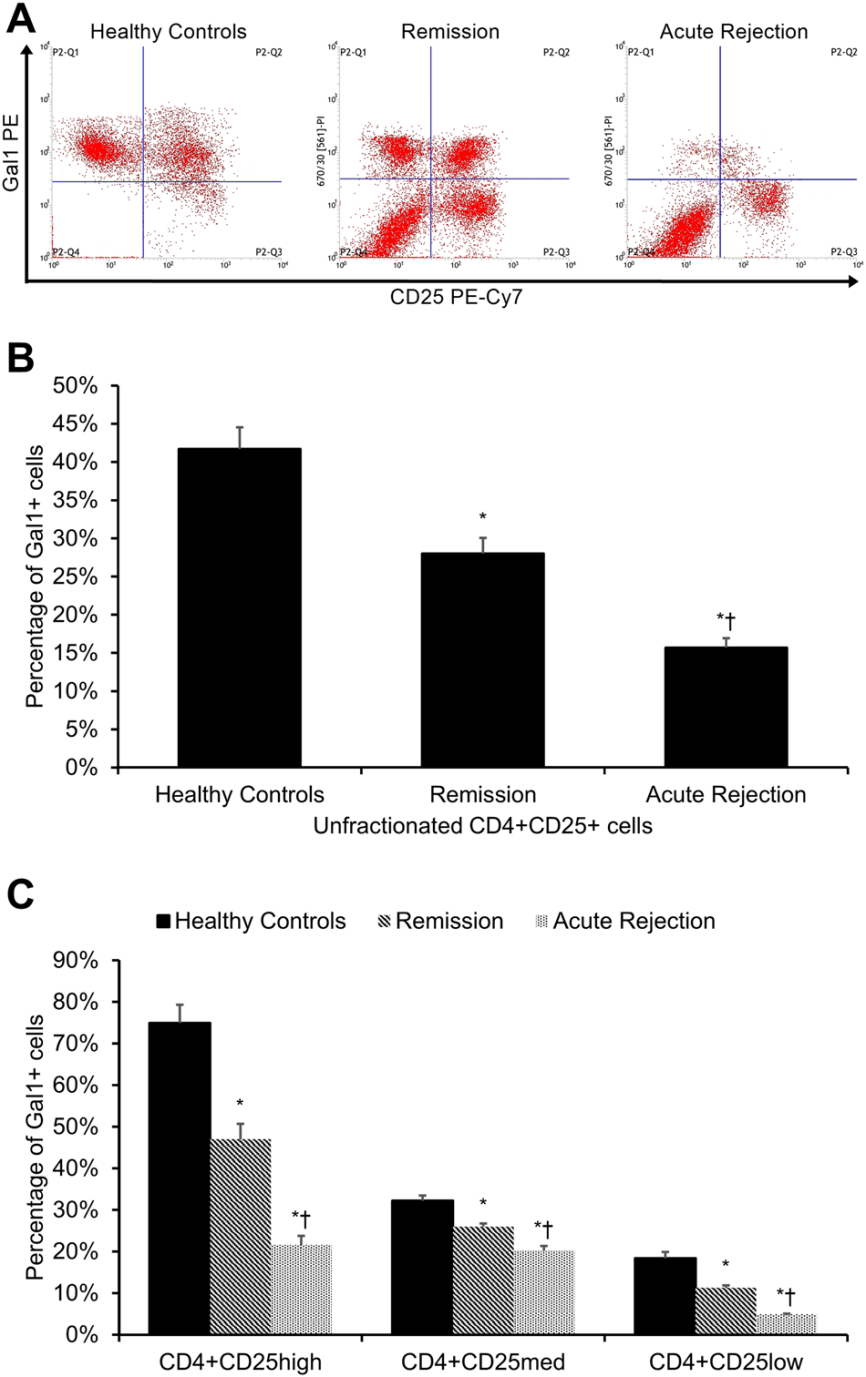
Percentage of Gal1+ regulatory T cells. **a** Representative dot plots of CD25 PE-Cy7 (*x*-axis) vs. Gal1 PE (*y*-axis) from a healthy control participant (left panel), a transplant patient in remission (middle panel), and an acute rejection transplant patient (right panel). Cells were gated on CD4+ T cells. Percentage of Gal1+ cells **b** within unfractionated regulatory T cells (CD4+CD25+ cells) and **c** within the subsets of CD4+CD25_high_, CD4+CD25_med_, and CD4+CD25_low_ cells in healthy controls (*n* = 40), transplant patients in remission (*n* = 85), and acute rejection transplant patients (*n* = 31). Each experiment was performed in triplicate. Results are reported as means ± standard errors of mean (SEMs). *p < 0.05 vs. healthy control group, ^†^*p* < 0.05 vs. remission group

The percentage of CD4+CD25+Gal1+ T_regs_ within the overall CD4+CD25+ T_reg_ population was significantly lower in transplant patients relative to healthy control subjects (*p* < 0.05, Fig. 2), with the CD4+CD25+Gal1+ T_regs_ percentage being lower in acute rejection transplant patients as compared to transplant patients in remission (*p* < 0.05, Fig. 2a, b). This observed difference was also significantly evident when the CD4+CD25+Gal1+ T_reg_ population was analyzed by CD25 expression, with Gal1+CD25_low_, Gal1+CD25_med_, and Gal1+CD25_high_ subsets all displaying the same trend (all *p* < 0.05, Fig. 2a, c).

Relative to healthy control subjects, CD4+CD25+Gal1+ T_regs_ in acute rejection transplant patients and transplant remission patients contained significantly higher proportions of IFNγ+ and IL-17+ cells (both *p* < 0.05, Table 3). In contrast, CD4+CD25+Gal1− T_regs_ in acute rejection transplant patients and transplant remission patients contained significantly higher proportions of RORC+, IFNγ+, and IL-17+ cells (all *p* < 0.05, Table 3) as well as a significantly lower proportion of FOXP3+ cells (*p* < 0.05, Table 3) relative to healthy control subjects. Compared with CD4+CD25+Gal1− T_regs_, CD4+CD25+Gal1+ T_regs_ in all three patient cohorts contained significantly higher proportions of GATA-3+, FOXP3+, IL-10+, and TGFβ+ cells (all *p* < 0.05, Table 3) as well as a significantly lower proportions of RORC+ and IL-17+ cells (both *p* < 0.05, Table 3).

**Table 3:** Cell subtype percentages within the CD4+CD25+Gal1+ and CD4+CD25+Gal1− cell populations

	**Gal1+**			**Gal1−**		
	**Acute Rejection**	**Remission**	**Healthy Controls**	**Acute Rejection**	**Remission**	**Healthy Controls**
T-bet+	8.3 ± 1.1	8.2 ± 1.1	8.1 ± 3.8	7.5 ± 1.2	6.7 ± 1.0	5.7 ± 1.1
GATA-3+	7.1 ± 1.2^‡^	7.2 ± 1.2^§^	7.3 ± 3.7^¶^	4.0 ± 1.1^‡^	4.0 ± 1.1^§^	4.0 ± 0.7^¶^
RORC+	0.9 ± 0.5^‡^	0.8 ± 0.4^§^	0.6 ± 0.3^¶^	7.7 ± 0.8*^‡^	5.5 ± 0.6*^§^	3.1 ± 0.6^¶^
FOXP3+	11.6 ± 1.6*^†‡^	34.7 ± 4.8^§^	60.3 ± 4.4^¶^	1.1 ± 0.2*^†‡^	6.1 ± 1.2*^§^	11.6 ± 1.1^¶^
IFNγ+	33.3 ± 4.5*	23.3 ± 3.2*	12.3 ± 1.8	48.6 ± 6.0*^†^	32.1 ± 3.9*	14.0 ± 2.5
IL-17+	1.9 ± 0.3*^†‡^	1.3 ± 0.2*^§^	0.6 ± 0.1^¶^	7.8 ± 1.2*^†‡^	4.9 ± 0.7*^§^	1.6 ± 0.5^¶^
IL-10+	4.1 ± 0.5*^†‡^	8.4 ± 1.0^§^	13.0 ± 0.7^¶^	1.2 ± 0.1*^†‡^	1.8 ± 0.2^§^	2.3 ± 0.3^¶^
TGFβ+	5.6 ± 0.6^‡^	6.9 ± 0.8^§^	8.4 ± 0.4^¶^	3.6 ± 0.3^‡^	3.8 ± 0.4^§^	4.0 ± 0.8^¶^

Data reported as means ± SDs

Within the Gal1+ subset or Gal1− subset: *p < 0.05 vs. healthy control group, ^†^*p* < 0.05 vs. remission group

Within the acute rejection group: ^‡^*p* < 0.05, Gal1+ subset vs. Gal1− subset

Within the remission group: ^§^*p* < 0.05, Gal1+ subset vs. Gal1− subset

Within the healthy control group: ^¶^*p* < 0.05, Gal1+ subset vs. Gal1− subset

### Responder T-cell proliferation and responsiveness to T_reg_ control

**Fig. 3:**
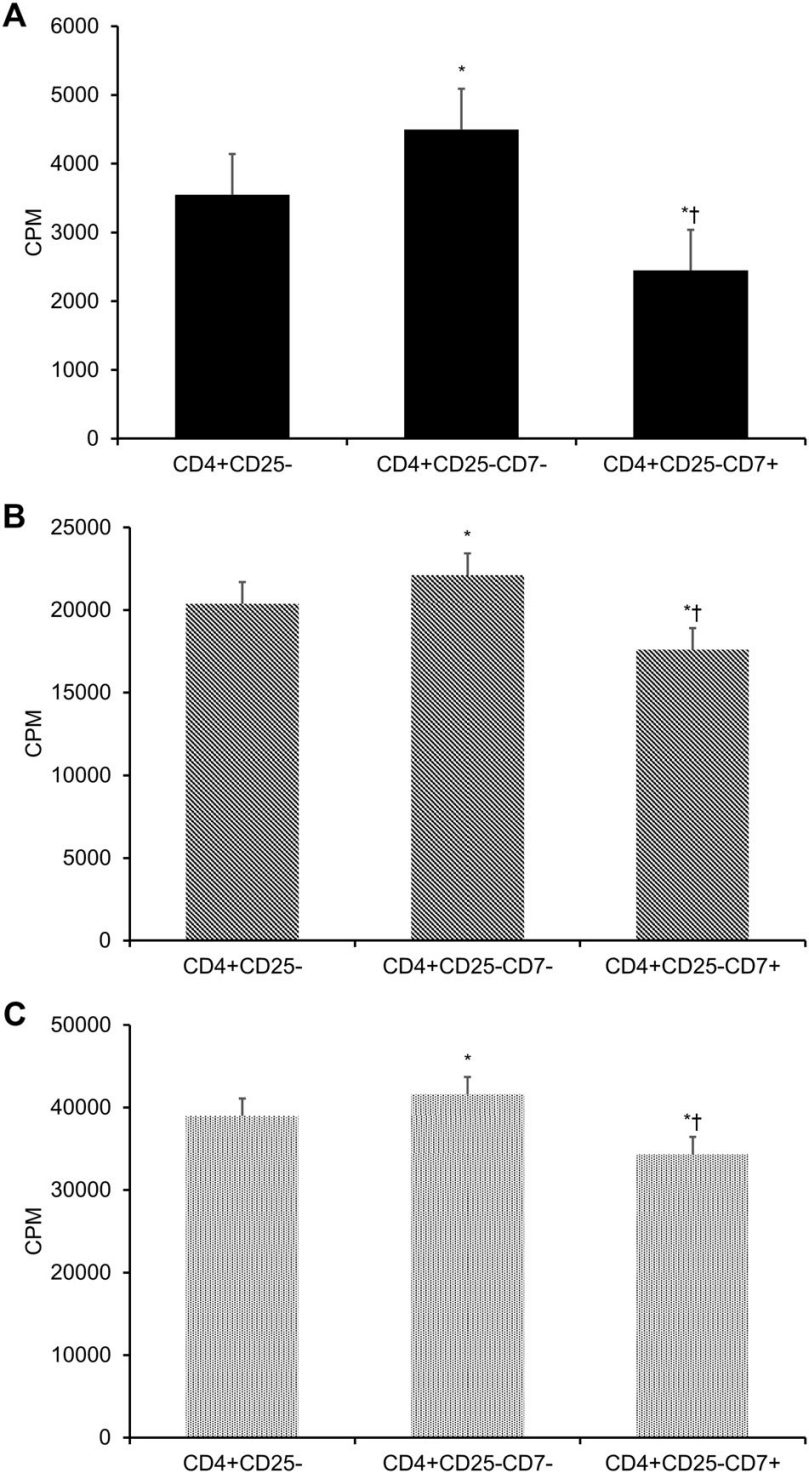
Proliferation of CD4+CD25−CD7− and CD4+CD25−CD7+ responder T cells. Two-step negative selection via immunomagnetic beads was applied to isolate CD4+CD25− cells from PBMCs. Then, the CD7− and CD7+ cell fractions were obtained therefrom after incubating the CD4+CD25− cells with a PE-labeled CD7 antibody and anti-PE microbeads. The unfractionated CD4+CD25− cells, the CD4+CD25−CD7− subset, and the CD4+CD25−CD7+ subset from (**a**) acute rejection transplant patients (*n* = 31), **b** transplant patients in remission (*n* = 85), and **c** healthy controls (*n* = 40) were cultured with CD3/CD28 T-cell expander and recombinant IL-2 over a period of 5 days. Each experiment was performed in triplicate. Results are reported as means ± standard errors of mean (SEMs). *p < 0.05 vs. CD4+CD25− group, ^†^*p* < 0.05 vs. CD4+CD25−CD7− group

**Fig. 4:**
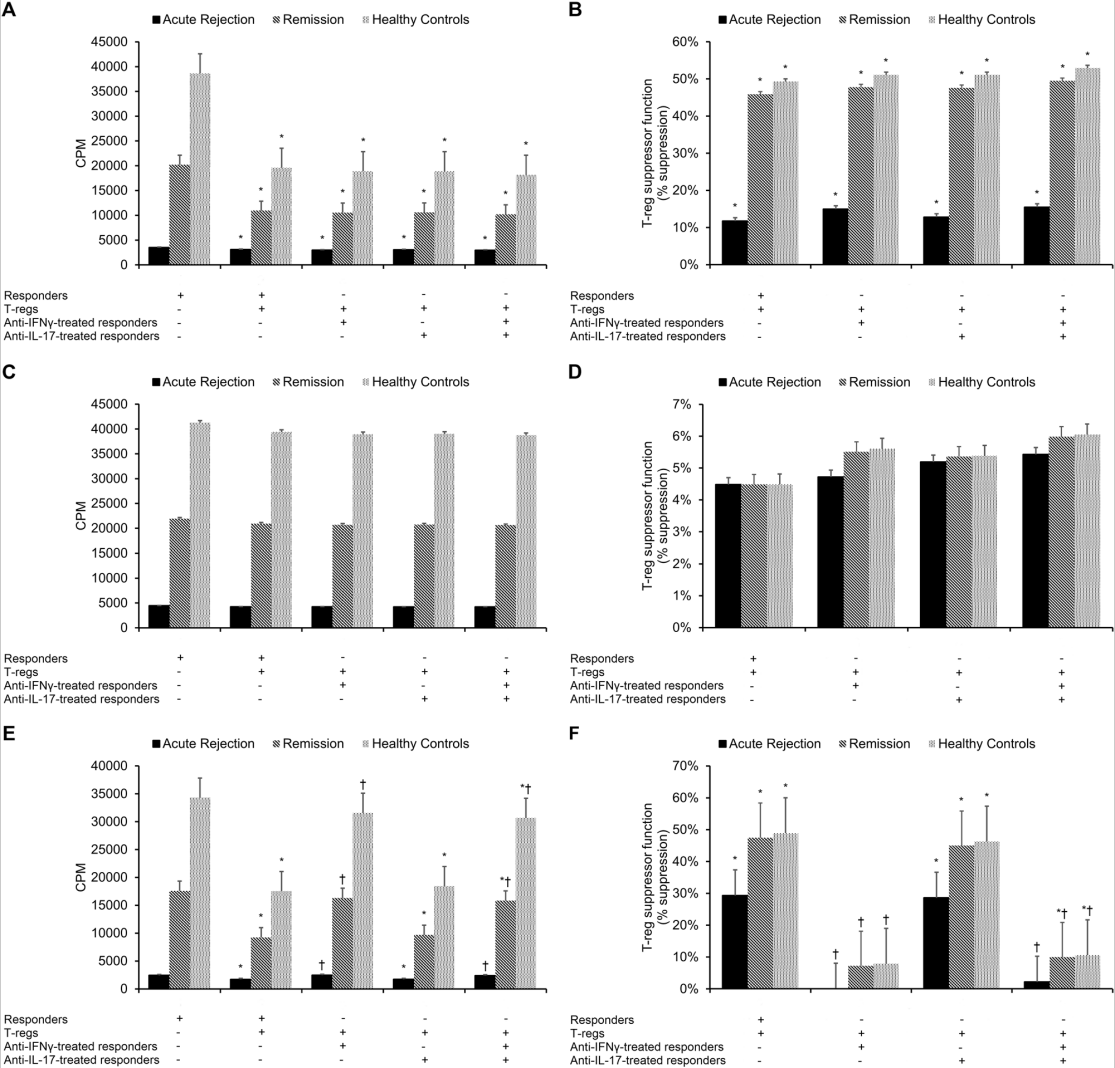
Effects of IFNγ and IL-17 on regulatory T-cell suppression of responder T cells. Unfractionated regulatory T cells (CD4+CD25+ cells) isolated from acute rejection transplant patients (*n* = 31), transplant patients in remission (*n* = 85), and healthy control subjects (*n* = 40) were added to **a**, **b** CD4+CD25−, **c**, **d** CD4+CD25−CD7−, or **e**, **f** CD4+CD25−CD7+ responder T cells, which were either left untreated or treated with neutralizing antibodies for anti-IFNγ, anti-IL-17, or anti-IFNγ + anti-IL-17. After a 5-day co-culturing period, ^3^H-thymidine incorporation was applied to measure **a**,** c**,** e** responder T-cell proliferation and **b**,** d**,** f** % suppression of responder T-cell proliferation (a measure of regulatory T-cell suppressor function). Each experiment was performed in triplicate. Results are reported as means ± standard errors of mean (SEMs). *p < 0.05 vs. responders only, ^†^*p* < 0.05 vs. responders + T_regs_

Across all three patient groups, the proliferation of the overall CD4+CD25− responder T-cell population was significantly higher than that of the CD4+CD25−CD7+ responder T-cell subset (*p* < 0.05, Fig. 3a–c) and significantly lower than that of CD4+CD25−CD7− responder T-cell subset (*p* < 0.05, Fig. 3a–c). The addition of unfractionated CD4+CD25+ T_regs_ significantly reduced responder T-cell proliferation by 12% in acute rejection transplant patients, 46% in transplant patients in remission, and 49% in healthy control subjects when unfractionated CD4+CD25− responder T cells were used (all *p* < 0.05, Fig. 4a, b) and by 29, 47, and 49% when the CD4+CD25−CD7+ responder T-cell subset were used (all *p* < 0.05, Fig. 4, f). The addition of unfractionated CD4+CD25+ T_regs_ did not significantly affect responder T-cell proliferation when the CD4+CD25−CD7− responder T-cell subset was used (*p* > 0.05, Fig. 4c, d).

As the proportions of IFNγ-expressing and IL-17-expressing cells were significantly greater in CD4+CD25+ T_regs_ from transplant patients as compared to those from healthy controls (Table 3), we next assessed the effect of neutralizing IFNγ and IL-17 on T_regs_ control of unfractionated CD4+CD25− responder T cells, the CD4+CD25−CD7− responder T-cell subset, and the CD4+CD25−CD7+ responder T-cell subset (Fig. 4). Neutralizing IFNγ failed to affect the responsiveness of CD4+CD25− responder T cells and CD4+CD25−CD7− responder T cells to T_reg_ control (*p* > 0.05, Fig. 4a–d). However, neutralizing IFNγ significantly reduced the responsiveness of CD4+CD25−CD7+ responder T cells to T_reg_ control (*p* < 0.05, Fig. 4e, f). Neutralizing IL-17 did not significantly affect the responsiveness of CD4+CD25− responder T cells, CD4+CD25−CD7− responder T cells, or CD4+CD25−CD7+ responder T cells to T_reg_ control in either transplant patients or healthy control subjects (Fig. 4a–f). Neutralizing both IFNγ and IL-17 significantly reduced the responsiveness of CD4+CD25−CD7+ responder T cells to T_reg_ control (*p* < 0.05, Fig. 4e, f) but did not significantly affect the responsiveness of CD4+CD25− responder T cells and CD4+CD25−CD7− responder T cells (Fig. 4a–d).

To further investigate whether IL-10 secretion influences T_reg_ control over responder T-cell proliferation, we next assessed the effect of neutralizing IL-10 on T_reg_ control of unfractionated CD4+CD25− responder T cells, the CD4+CD25−CD7− responder T-cell subset, and the CD4+CD25−CD7+ responder T-cell subset (Supplementary Figure 1). Neutralizing IL-10 failed to affect the responsiveness of CD4+CD25− responder T cells and CD4+CD25−CD7− responder T cells to T_reg_ control (*p* > 0.05, Supplementary Figure 1A–D). However, neutralizing IL-10 significantly reduced the responsiveness of CD4+CD25−CD7+ responder T cells to T_reg_ control (*p* < 0.05, Supplementary Figure 1E and Supplementary Figure 1F).

### Effect of Gal1 silencing on T_regs_

Application of Gal1-siRNA to T_regs_ significantly decreased Gal1 mRNA expression by 86% in acute rejection transplant patients, by 87% in transplant patients in remission, and by 89% in healthy control subjects (all *p* < 0.05, Supplementary Figure 2A). Application of Gal1-siRNA to T_regs_ significantly decreased Gal1 protein expression by 92% in acute rejection transplant patients, by 92% in transplant patients in remission, by 94% in healthy control subjects (all *p* < 0.05, Supplementary Figure 2B).

**Fig. 5:**
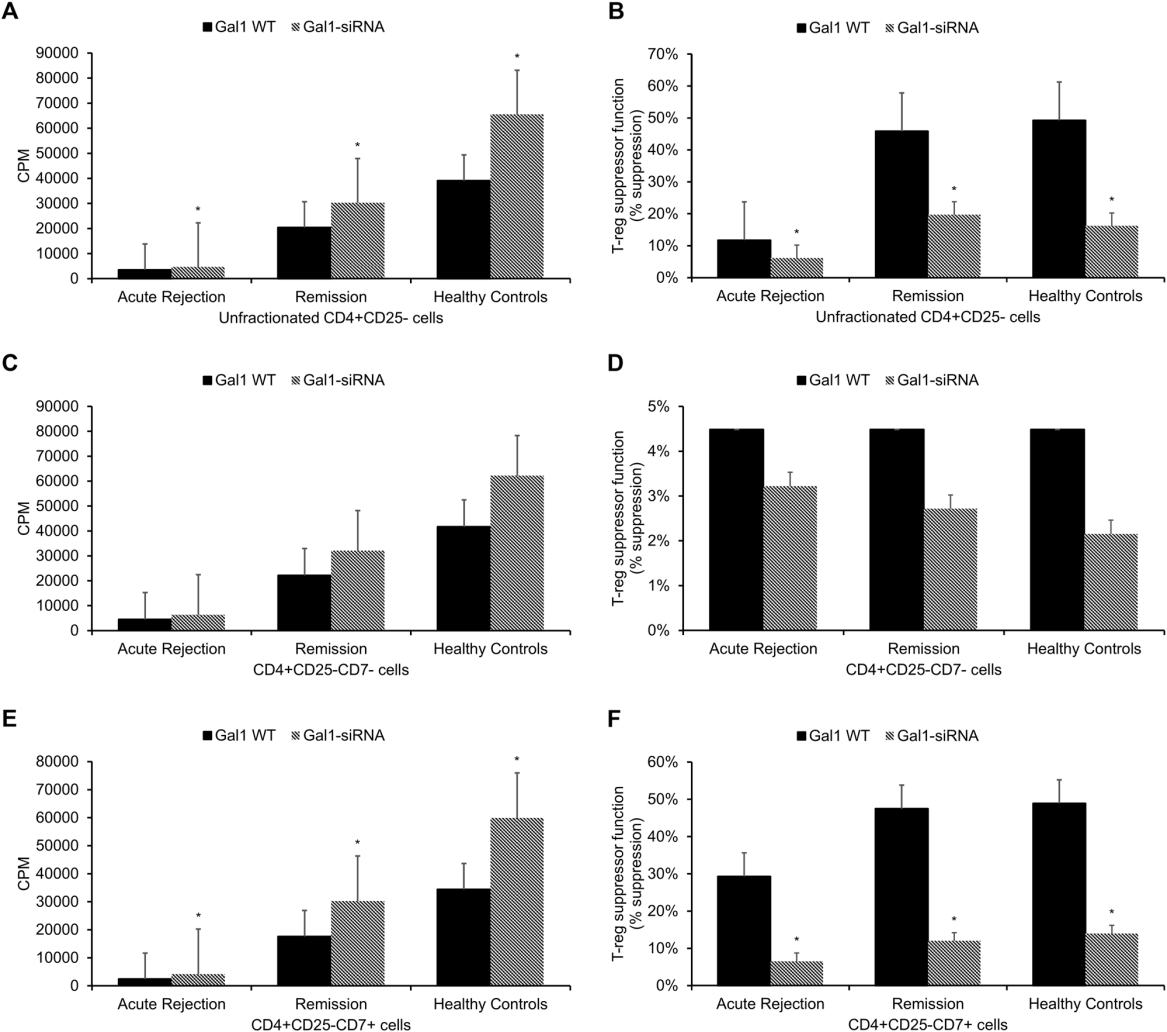
Effect of Gal1 silencing on regulatory T-cell suppression of responder T cells. Gal1 WT and Gal1-silenced regulatory T cells (CD4+CD25+ cells) isolated from acute rejection transplant patients (*n* = 31), transplant patients in remission (*n* = 85), and healthy control subjects (n = 40) were added to **a**, **b** CD4+CD25−, **c**, **d** CD4+CD25−CD7−, or **e**, **f** CD4+CD25−CD7+ responder T cells. After a 5-day co-culturing period, ^3^H-thymidine incorporation was applied to measure **a**,** c**,** e** responder T-cell proliferation and **b**,** d**,** f** % suppression of responder T-cell proliferation (a measure of regulatory T-cell suppressor function). Each experiment was performed in triplicate. Results are reported as means ± standard errors of the mean (SEMs). *p < 0.05 vs. Gal1 WT group

Gal1 silencing of T_regs_ significantly reduced the inhibition of responder T-cell proliferation from 12 to 6% in acute rejection transplant patients, from 46 to 20% in transplant patients in remission, and from 49 to 16% in healthy control subjects when CD4+CD25− responder T cells were used (all *p* < 0.05, Fig. 5a, b), and from 29 to 7% in acute rejection transplant patients, from 47 to 12% in transplant patients in remission, and from 49 to 14% in healthy control subjects when CD4+CD25−CD7+ responder T cells were used (all *p* < 0.05, Fig. 5e, f). Gal1 silencing of T_regs_ did not significantly affect responder T-cell proliferation when CD4+CD25−CD7− responder T cells were used (*p* > 0.05, Fig. 5c, d).

### Analysis of CD43 and CD45 expression on responder T cells

Having demonstrated that T_reg_ Gal1 expression plays a critical suppressive role upon CD4+CD25−CD7+ responder T cells, we next analyzed the expression of two other glycoproteins—CD43 and CD45—that have been shown to regulate Gal1 binding and responder T-cell susceptibility to Gal1-induced apoptosis^9^. Consistent with our CD7 findings (Fig. 1), we found that the proportions of CD43+ responder T cells and CD45+ responder T cells within the overall CD4+CD25− responder T-cell population were significantly lower in acute rejection transplant patients relative to healthy control subjects (*p* < 0.05, Supplementary Figure 3A–D). Moreover, there were significant positive correlations between CD43 expression and CD7 expression (*r* = 0.76, *p* < 0.05, Supplementary Figure 3E) as well as between CD45 expression and CD7 expression (*r* = 0.81, *p* < 0.05, Supplementary Figure 3F) on CD4+CD25− responder T cells.

In order to better understand the effects of CD43 and CD45 expression upon CD4+CD25−CD7+ responder T-cell apoptosis, we next re-analyzed apoptosis levels in CD7+ responder T cells by segregating these responder T cells into four groups: CD7+CD43_high_ responder T cells; CD7+CD43_low_ responder T cells; CD7+CD45_high_ responder T cells; and CD7+CD45_low_ responder T cells. We found that CD7+CD43_high_ responder T cells showed significantly higher apoptosis levels relative to CD7+CD43_low_ responder T cells in all three patient cohorts (*p* < 0.05, Supplementary Figure 4A). The accompanying correlation analysis revealed a significant positive correlation between CD43 expression and CD7+ responder T-cell apoptosis levels (*r* = 0.56, *p* < 0.05, Supplementary Figure 4B). However, there were no significant differences in apoptosis levels between CD7 + CD45_high_ and CD7+CD45_low_ responder T cells across all three cohorts (*p* < 0.05, Supplementary Figure 4C) and no significant correlation between CD45 expression and CD7+ responder T-cell apoptosis levels (*r* = 0.15, *p* > 0.05, Supplementary Figure 4D).

Having shown that CD7+CD43_high_ responder T cells displayed significantly higher apoptosis levels relative to CD7+CD43_low_ responder T cells, we next investigated whether this effect is Gal1-dependent. Through Gal1-siRNA transfection of T_regs_, we found that the pro-apoptotic effect of high CD43 expression on CD7+responder T cells was abrogated by Gal1 silencing (*p* < 0.05, Supplementary Figure 2E). These combined findings suggest that CD43 co-expression on CD7+responder T cells promotes their apoptosis in a Gal1-dependent manner.

### Clinical correlation analysis

The CD7+_all_ cell percentage was significantly and inversely correlated with AST, ALT, and GGT levels (*r* = −0.46, −0.45, and −0.47, all *p* < 0.05; Supplementary Table 1). Notably, the CD7+CD43_high_ and CD7+CD45_high_ cell percentages were more strongly correlated with AST, ALT, and GGT levels (*r* = −0.61, −0.59, and −0.62 for CD7+CD43_high_; −0.86, −0.84, and −0.89 for CD7+CD45_high_, all *p* < 0.05; Supplementary Table 1). Conversely, the CD7−T-bet+ cell percentage was significantly and positively correlated with AST, ALT, and GGT levels (*r* = 0.67, 0.66, and 0.69, all *p* < 0.05; Supplementary Table 1) and negatively correlated with the CD7+_all_ cell percentage (*r* = −0.91, *p* < 0.05; Supplementary Table 1).

## Discussion

This study demonstrates that in liver allograft rejection, inhibition of T_reg_ function is associated with decreased responder T-cell susceptibility to T_reg_ control, indicating defects at both the T_reg_ and effector T-cell levels. Therefore, this study validates the critical role of proper T_reg_ function in allograft tolerance^10^ and also shows that Gal1 downregulation may be a key mechanism underlying T_reg_ inhibition in acute allograft rejection, as Gal1-silenced T_reg__s_ were less efficacious at controlling responder T-cell proliferation.

Liver allograft rejection patients displayed CD7 downregulation on the surface of CD4+CD25− responder T cells accompanied by a reduced CD4+CD25+Gal1+ T_reg_ percentage. However, the mechanism(s) underlying this CD7 downregulation on responder T cells remains unknown. That being said, our current observations lend support to a dysfunctional Th1 cell differentiation process, as we observed a heightened T-bet+ cell percentage on CD7− responder T cells in liver allograft rejection patients. As T-bet has been shown to induce the expression of several Th1 surface markers (e.g., CD7, CD26, CD27, IL-18R, and IL-18RAcP)^11^, our findings suggest that in liver allograft rejection patients, CD4+ Th1 cells show a dysfunctional tendency to arrest at the CD7−T-bet+ cell phenotype without fully differentiating into the CD7+T-bet+ responder T-cell phenotype that is susceptible to Gal1+ T_reg_ control via Gal1/CD7 binding^12^.

In addition, here we clearly demonstrated that CD7 expression renders responder T cells responsive to T_reg_ control through a set of experiments in which CD7− and CD7+ responder T-cell subsets were separately assessed for their responsiveness to T_reg_ control. Specifically, the CD7+ responder T-cell subset was the most sensitive to T_reg_ control. Notably, CD7+ cell responsiveness was stronger in healthy subjects as compared to liver allograft rejection patients, indicating that liver allograft rejection patients display decreased responder T-cell responsiveness to T_reg_ suppression as well as dysfunctional T_reg_ function. Therefore, the increase in CD7−T-bet+ responder T cells in liver allograft rejection patients may represent a poorly controlled population of responder T cells that promote alloreactive hepatic injury. This conclusion is supported by the positive correlation between the proportion of CD7−T-bet+ responder T cells and the degree of hepatic injury (as measured by serum ALT, AST, and GGT levels) as well as the negative correlation between the proportion of CD7+ responder T cells and the degree of hepatic injury. Similarly, the most common form of cutaneous T-cell lymphoma—mycosis fungoides—is characterized by focal plaques and tumors of poorly controlled malignant CD4+ T cells, in which CD7− status correlates with resistance to Gal1-induced apoptosis^13^. These combined findings suggest that restoring CD7 expression on responder T cells may ameliorate hepatic injury in liver allograft rejection patients through reinstating proper T_reg_ control.

Moreover, here we discovered elevated proportions of CD7+IFNγ+ and CD7+IL-17+ in liver allograft rejection patients, suggesting that Th1 and Th17 effector cells, respectively, may be involved in the responsiveness of responder T cells to T_reg_ control. On this basis, we next assessed whether regulation of responder T cells by T_reg__s_ is dependent upon IFNγ and/or IL-17 production. We found that antibody-based neutralization of IFNγ was able to significantly reduce the responsiveness of CD7+ responder T cells to T_reg_ control (but did not affect the responsiveness of CD7− responder T cells). These findings indicate that IFNγ specifically contributes to Gal1+ T_reg_ suppression of CD7+ responder T cells. These findings echo those of work by Liberal et al.^14^ on the Gal-9/Tim-3 pathway, which demonstrated that IFNγ specifically contributes to Gal-9+ T_reg_ suppression of Tim-3+ responder T cells. Although previous work has shown that Gal1 suppresses IFNγ secretion from CD4+ T cells^15,16^ as well as CD8+ T cells^17^, further research on IFNγ’s role in supporting Gal1+ T_reg_ regulation of CD7+ responder T cells is still needed.

As T_reg_-derived IL-10 has been shown to be critical in mediating tolerance to alloantigens^18^, suppressing naive CD4+ T-cell proliferation in immunocompromised models^19^, and controlling several autoimmune disorders^20–22^, we next assessed whether regulation of CD4+CD25− responder T cells by T_regs_ is dependent upon IL-10 production. We found that antibody-based neutralization of IL-10 was able to significantly reduce the responsiveness of CD7+ responder T cells to T_reg_ control (but did not affect the responsiveness of CD7− responder T cells). These findings indicate that IL-10 specifically contributes to Gal1+ T_reg_ regulation of CD7+ responder T cells. Accordingly, we also observed a significant decrease in the proportion of Gal1+IL-10+ T_reg__s_ in liver allograft rejection patients, which may represent a “weakened” population of Gal1+ T_reg__s_ that are not as effective at controlling CD7+ responder T cells. Interestingly, Cedeno-Laurent et al.^23^ has demonstrated that Gal1 binding to CD45 on Th cells promotes IL-10 production and that these Gal1-induced IL-10+ Th cells display a Tr1-like function by suppressing T-cell proliferation. Therefore, these findings suggest that Gal1+ T_reg__s_ can act through directly binding to CD7+ responder T cells as well as through promoting the differentiation of suppressive IL-10+ Th cells.

In addition to CD7, it is well-established that Gal1 binds to the glycoproteins CD43 and CD45 on responder T cells^9^. These glycoproteins are involved in regulating Gal1-induced apoptosis, as specific oligosaccharide modifications of CD43 and CD45 have been shown to affect Gal1 binding and T-cell susceptibility to Gal1-induced apoptosis^9^. Notably, mirroring the CD7+ responder T-cell data, we found that acute rejection patients displayed significantly lower proportions of CD43+ and CD45+ responder T cells relative to healthy controls. More interestingly, we found that CD43 co-expression (but not CD45 co-expression) on CD7+ responder T cells promotes their apoptosis in a Gal1-dependent manner. These findings concord with study by Pericolini et al.^24^, which demonstrated that CD7 and CD43 mediate human T-cell apoptosis, while CD45 solely mediates immortalized Jurkat cell apoptosis. These combined findings support the contention that CD43 is a potentiator of Gal1/CD7-mediated T-cell apoptosis and that responder T-cell CD43 downregulation in acute rejection patients may further contribute to reduced responder T-cell responsiveness to T_reg_ control.

In terms of potential clinical applications, the current results can be applied to construct a T_reg_-based immunotherapeutic regimen for liver allograft rejection patients. Specifically, as Gal1 plays a key role in maintaining optimal T_reg_ function in humans^25^, researchers should investigate whether proper T_reg_ suppression in liver allograft rejection patients can be restored through cell culturing in a Gal1-positive environment and/or transduction with Gal1 cDNA. Indeed, culturing of peripheral blood mononuclear cells (PBMCs) from human leukocyte antigen-mismatched donors in the presence of recombinant Gal1 has been shown to induce dose-dependent inhibition of the allogenic T-cell response through Gal1-driven apoptosis of activated T cells^26^. Moreover, in a rheumatoid arthritis murine model, syngeneic fibroblasts genetically engineered to produce Gal1 have been shown to suppress the autoimmune response through increasing activated T-cell apoptosis^27^.

In conclusion, our findings demonstrate that dysfunctional immunoregulation in liver allograft rejection patients is attributable (in part) to the failure of adequate T_reg_ suppression on account of Gal1 downregulation as well as a reduced responsiveness of responder T cells to T_reg_ control on account of CD7 downregulation. Responder T-cell CD43 downregulation in acute rejection patients may further contribute to reduced responder T-cell responsiveness to T_reg_ control. As CD7 and CD43 expression are more downregulated during active rejection, the adoptive transfer of autologous T_reg__s_ may be an effective strategy for targeting effector T cells.

## Materials and methods

### Ethics statement

The protocols of this study were reviewed and approved by the Ethics Committee of the People’s Hospital of Zhengzhou (no. 2015106, Zhengzhou, China). Written informed consent was obtained from each participant prior to inclusion.

### Patient recruitment

Adult liver transplant candidates (18 years of age and older) were prospectively screened at the Department of Hepatobiliary Surgery at People’s Hospital of Zhengzhou. All liver transplant candidates received intravenous methylprednisolone (1.0 g) along with oral mycophenolate (1.5 g) immediately prior to liver transplantation. Following liver transplantation, maintenance immunosuppression was administered consisting of cyclosporine or tacrolimus, mycophenolate mofetil (1.0 g twice daily according to WBC count), and tapered prednisone dosing over a 3-month period. Calcineurin inhibitors were added on post-operative day 3.

Candidates were followed-up in the outpatient clinic on a weekly basis for the first month, on a biweekly basis for the second and third months, monthly up to 1 year, and subsequently every 3 months under the discretion of the attending physician. Candidates were screened for acute cellular rejection (hereinafter acute rejection) through a combination of clinical examination and liver function testing, and the preliminary diagnosis of acute rejection was then validated via transjugular liver biopsy using the Banff criteria. Candidates with a diagnosis of chronic rejection, hepatic viral infections (e.g., HBV and HCV), or autoimmune hepatitis were excluded. After screening, 116 liver transplant patients were finally recruited into this study.

All acute rejection liver transplant patients were investigated while actively on immunosuppressive therapy, as T_regs_ sampled at disease presentation prior to immunosuppression have been demonstrated to be inefficient at cell proliferation suppression. Immunosuppressive therapy consisted of intravenous methylprednisolone (250 mg) for 3 days, followed by tapered dosing. Patients underwent a follow-up liver biopsy 2 weeks after initiation of immunosuppressive therapy to assess their therapeutic response. Transplant patients that failed to respond to immunosuppressive therapy were placed in the “acute rejection” group (*n* = 31), while those that entered remission following immunosuppressive therapy were placed in the “remission” group (*n* = 85). In addition, 40 adult healthy participants were prospectively recruited from the same hospital to serve as healthy controls. Demographic and clinical data for the participants are detailed in Table 1.

### PBMC preparation

PBMCs were collected as previously described^14^. The viability of PBMCs was assessed using trypan blue exclusion with a 98% threshold. Cryopreserved PBMCs were stored within liquid nitrogen till later testing. PBMC preparations from test case participants were tested prior to and following cryopreservation to assure no significant differences in cellular viability, proliferation, cytokine production, and suppressive characteristics.

### Flow cytometry

Flow cytometry was performed as previously described with minor modifications^14^. Briefly, unfractionated PBMCs were primarily stained with the following antibodies (BD Biosciences Discovery Labware, Bedford, MA, USA): anti-CD4 (allophycocyanin (APC)-cychrome-(Cy)-7-conjugated), anti-CD25 (fluorescein isothiocyanate (FITC)-conjugated or phycoerythrin (PE)-Cy7-conjugated), anti-CD7 (PE- or APC-conjugated), anti-CD43 (PE-conjugated), and anti-CD45 (PE-conjugated). These cells were then incubated at 4 °C in a dark room for ~30 min, and then washed with a solution containing phosphate-buffered saline and 1% fetal bovine serum. After being washed, cells were then re-suspended and analyzed by fluorescent-activated cell sorting (FACS) using a Becton Dickinson instrument (San Jose, CA, USA), and the FACSDiva package was employed to perform the data analysis. At least 2 × 10^4^ gated events were gathered per sample. Experiments were performed in triplicate.

The T-bet+, GATA-3+, RORC+, and FOXP3+ cell percentages (for Th1 cells, Th2 cells, Th17 cells, and T_regs_, respectively) were identified by fixation, permeabilization (Cytofix/Cytoperm, BD Biosciences Discovery Labware), and counterstaining with anti-T-bet (peridinin chlorophyll protein (PCP)-Cy5-conjugated), anti-GATA-3 (PE-Cy7-conjugated), anti-RORC (PE-conjugated), or anti-FOXP3 (FITC-conjugated), respectively (BD Biosciences Discovery Labware). Incubation using murine anti-human Gall along with a PE-conjugated secondary antibody (Invitrogen, Carlsbad, CA, USA) was employed to assess the overall percentage of Gall+ cells. Experiments were performed in triplicate.

IFNγ-producing, IL-10-producing, IL-17-producing, and transforming growth factor-beta (TGFβ)**-**producing cells were analyzed prior to and following incubation with ionomycin (500 ng/ml) and phorbol 12-mystrate 13-acetate (10 ng/ml; Sigma Aldrich, St. Louis, MO, USA), brefeldin A (10 µg/ml for 5 h, Sigma Aldrich), and counterstaining with anti-IFNγ, anti-IL-10, anti-IL-17 (FITC- or APC-conjugated), and anti-TGF-β (PCP-conjugated; BD Biosciences Discovery Labware). Flow cytometry was then conducted as described above. Experiments were performed in triplicate.

### Cell isolation and purification

CD4+CD25− cells and CD4+CD25+ cells (hereinafter T_regs_) were isolated from the PBMC population with the help of immunomagnetic beads as previously described^14^. CD4+CD25− cells were further analyzed and purified using CD7 expression. In short, CD4+CD25− cells were treated with anti-CD7 (PE-conjugated) for 30 min followed by incubation with anti-PE antibody-conjugated microbeads (Miltenyi Biotec, Bergisch-Gladbach, Germany) for 15 min (4 °C). Then, the resulting CD4+CD25−CD7+ cells and CD4+CD25−CD7− cells were purified by positive and negative selection, respectively, with MS separation columns (Miltenyi Biotec). CD4+CD25−CD7+ cells were further analyzed and sorted according to their levels of CD43 and CD45 expression (i.e., CD43_low_, CD43_high_, CD45_low_, and CD45_high_).

Similarly, CD4+CD25+ cells were treated with anti-Gal1 (PE-conjugated) for 30 min followed by incubation with anti-PE antibody-conjugated microbeads (Miltenyi Biotec) for 15 min (4 °C). Then, the resulting CD4+CD25+Gal1+ cells and CD4+CD25+Gal1− cells were purified by positive and negative selection, respectively, with MS separation columns (Miltenyi Biotec) and were further analyzed and sorted according to their levels of CD25 expression (i.e., CD25_low_, CD25_med_, and CD25_high_).

### Cell proliferation assays

Cell proliferation of responder T cells was assayed as previously described^14^. After purification, T_regs_ were added to autologous CD4+CD25− cells, CD4+CD25−CD7+ cells, or CD4+CD25−CD7− cells (T_reg_: responder T-cell ratio of 1:84). Cells were cultured (5% CO_2_, 37 °C) with anti-CD3 and anti-CD28 T-cell expander (bead: cell ratio of 1:2; Dynal Biotech, Oslo, Norway) along with recombinant IL-2 (30 U/ml; Peprotech, NJ, USA) for 5 days. Similarly, CD4+CD25− cells, CD4+CD25−CD7+ cells, and CD4+CD25−CD7− cells were cultured without T_regs_ under identical conditions for control purposes. Over the final 18 h, these cells were pulsed with 3H-thymidine (0.25 μCi/well) and then harvested with the help of a multichannel harvester. The amount of cellularly incorporated 3H-thymidine was assessed with a β-counter (Perkin Elmer, Shelton, CT, USA). The proliferative inhibition percentage was calculated as follows: (1 − CPM [with T_regs_]) ÷ (CPM [without T_regs_]). Experiments were performed in triplicate.

### Neutralization assays

Neutralization assays were performed as previously described^14^. In order to assess whether the responder T cell’s susceptibility to T_reg_ control is associated with IFNγ or IL-17 secretion, CD4+CD25− cells, CD4+CD25−CD7+ cells, and CD4+CD25−CD7− cells were exposed to anti-IFNγ- or anti-IL-17-neutralizing antibodies (10 μg/ml; BD Biosciences Discovery Labware) for 12 h prior to the addition of T_regs_ as well as during the 5-day co-culture period. In order to assess whether the responder T cell’s susceptibility to T_reg_ control is associated with IL-10 secretion, CD4+CD25− cells, CD4+CD25−CD7+ cells, and CD4+CD25−CD7− cells co-cultured with T_regs_ were exposed to anti-IL-10-neutralizing antibodies (10 μg/ml; BD Biosciences Discovery Labware). Experiments were performed in triplicate.

### Gal1 silencing

In order to assess whether Gall expression affects T_regs_’ ability to suppress responder T cells, T_regs_ were incubated with three anti-Gal1 Stealth RNAis (Gal1-siRNA; Invitrogen) to silence Gal1 expression as previously described with minor modifications^14^. Opti-MEM medium (Invitrogen) was used to re-suspend the cells (2.0–3.0 × 10^6^/ml). Lipofectamine RNAiMAX reagent (Invitrogen) was applied to transfect the Gal1-siRNA (3 nM). A positive control glyceraldehyde 3-phosphate dehydrogenase Stealth RNAi (Invitrogen) and a negative control Stealth RNAi (Invitrogen) were used as transfection controls. After overnight incubation (5% CO_2_, 37 °C), two aliquots (2.5 × 10^5^ cells each) were employed to measure Gal1 mRNA and protein expression as detailed below. Following Gal1-siRNA transfection, T_regs_ were co-cultured with CD4+CD25− cells, CD4+CD25−CD7+ cells, and CD4+CD25−CD7− cells in order to assess Gal1’s effects on proliferation as described above. Experiments were performed in triplicate.

### Real-time quantitative reverse transcription-PCR

Quantitative reverse transcription was performed as previously described with minor modifications^14^. SuperScript™ First-Strand Synthesis System (Invitrogen) was employed to synthesize Oligo-dT-primed first-strand cDNA. The sequences used for PCR primers and internal probes were as follows: Gal1, 5′-TGAACCTGGGCAAAGACAGC-3′ (forward) and 5′-TTGGCCTGGTCGAAGGTGAT-3′ (reverse); β-actin, 5′-AGAAAATCTGGCACCACACC-3′ (forward) and 5′-CCATCTCTTGCTCGAAGTCC-3′ (reverse). Gal1 primers were applied at 300 and 400 nM concentrations, whereas the probe was applied at 200 nM. For the β-actin control, primers were applied at 20 and 70 nM, whereas the probe was applied at 100 nM. The other components of the PCR reaction were taken from the TaqMan Universal Master Mix (Applied Biosystems, Foster City, CA, USA). Using an ABI Prism 7700 instrument (Applied Biosystems), 45 cycles at 95° for 15 s and 60° for 60 s were performed. For each sample, relative mRNA levels were quantified with threshold cycle curves for Gal1 and β-actin. Experiments were performed in triplicate.

### Western blotting

Western blotting was performed as previously described with minor modifications^14^. Total protein was extracted and then separated by the use of 12% SDS-polyacrylamide gel electrophoresis, and then transferred to a polyvinylidene difluoride membrane. The membrane was placed in a 5% fat-free skimmed milk solution in Tris-buffered saline/0.05% Tween 20 for at least 1 h at room temperature, followed by an anti-Gal1 antibody (1:1000, Santa Cruz Biotechnology, Santa Cruz, CA, USA). Membranes were stripped and re-probed with an anti-β-actin antibody (Santa Cruz Biotechnology) to validate equalized lane loading. Membranes were treated with a horseradish peroxidase-linked secondary antibody (Santa Cruz Biotechnology) for 1 h at room temperature. The resulting bands were visualized using an enhanced chemiluminescence system (Pierce Biotechnology, Rockford, IL, USA). Experiments were performed in triplicate.

### Statistical analysis

Normality was assessed using the Kolmogorov–Smirnov goodness-of-fit test. Then, comparisons between experimental groups were performed using Student’s *t*-testing or one-way analysis of variance where appropriate. Pearson’s correlation was used to calculate the degree of linear relationship between variables. Results are reported as means ± SEMs from triplicate experiments. A *p*-value of <0.05 was deemed to be statistically significant.

## Electronic supplementary material

Supplementary Figure 1

Supplementary Figure 2

Supplementary Figure 3

Supplementary Figure 4

Supplementary Table 1

Summary of Supplementary Information
