# Supplementary material for: Dysfunctional immunoregulation in human liver allograft rejection associated with compromised galectin-1/CD7 pathway function
Source: Cell Death Dis. 2018 Feb 20;9(3):293. doi: 10.1038/s41419-017-0220-3 (PMC5833641; doi:10.1038/s41419-017-0220-3)

**Supplementary Figure 1. Effect of IL-10 on Regulatory T-Cell Suppression of Responder T-Cells**

Unfractionated regulatory T-cells (CD4+CD25+ cells) isolated from acute rejection transplant patients (n = 31), transplant patients in remission (n = 85), and healthy controls (n = 40) were added to (A) CD4+CD25-, (B) CD4+CD25-CD7-, or (C) CD4+CD25-CD7+ responder T-cells, which were either left untreated or treated with neutralizing antibodies for anti-IL-10. After a five-day co-culturing period, ^3^H-thymidine incorporation was applied to measure (A, C, E) responder T-cell proliferation and (B, D, F) % suppression of responder T-cell proliferation (a measure of regulatory T-cell suppressor function). Each experiment was performed in triplicate. Results are reported as means ± standard errors of the mean (SEMs). **P*<0.05 versus responders only, †*P*<0.05 versus responders + T-regs.


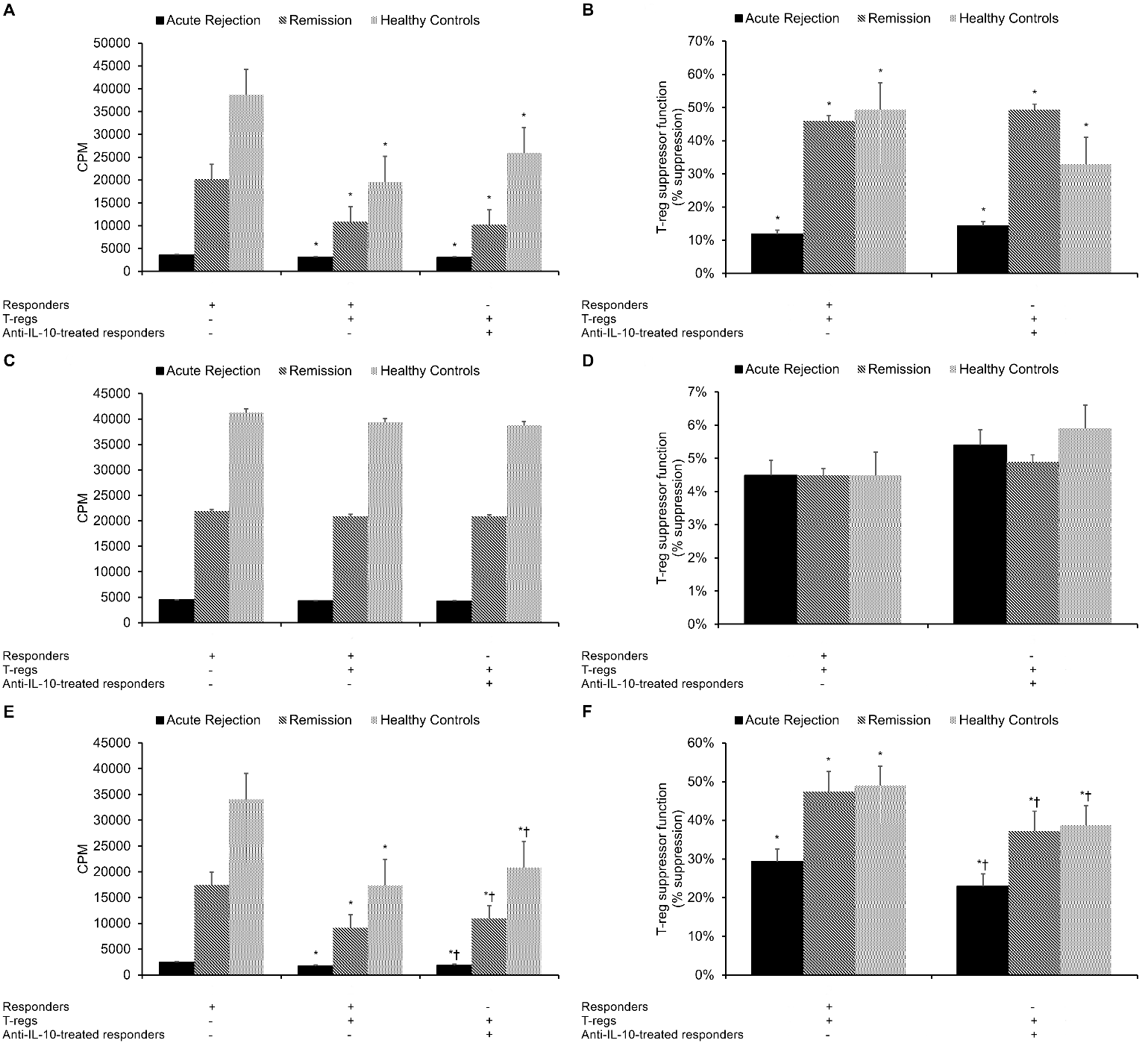

Supplement: Supplementary file 1 — Supplementary Figure 1 [file 41419_2017_220_MOESM1_ESM.docx]
