# Supplementary material for: Dysfunctional immunoregulation in human liver allograft rejection associated with compromised galectin-1/CD7 pathway function
Source: Cell Death Dis. 2018 Feb 20;9(3):293. doi: 10.1038/s41419-017-0220-3 (PMC5833641; doi:10.1038/s41419-017-0220-3)
Supplement: Supplementary file 2 — Supplementary Figure 2 [file 41419_2017_220_MOESM2_ESM.docx]

**Supplementary Figure 2. Validation of Gal1 Knockdown by Gal1 Silencing RNA (Gal1-siRNA)**

Unfractionated regulatory T-cells (CD4+CD25+ cells) isolated from acute rejection transplant patients (n = 31), transplant patients in remission (n = 85), and healthy controls (n = 40) treated with Gal1-siRNA displayed significant decreases in (A) Gal1 mRNA expression and (B) Gal1 protein expression. Each experiment was performed in triplicate. Results are reported as means ± standard errors of the mean (SEMs). **P*<0.05 versus Gal1 WT group.

**
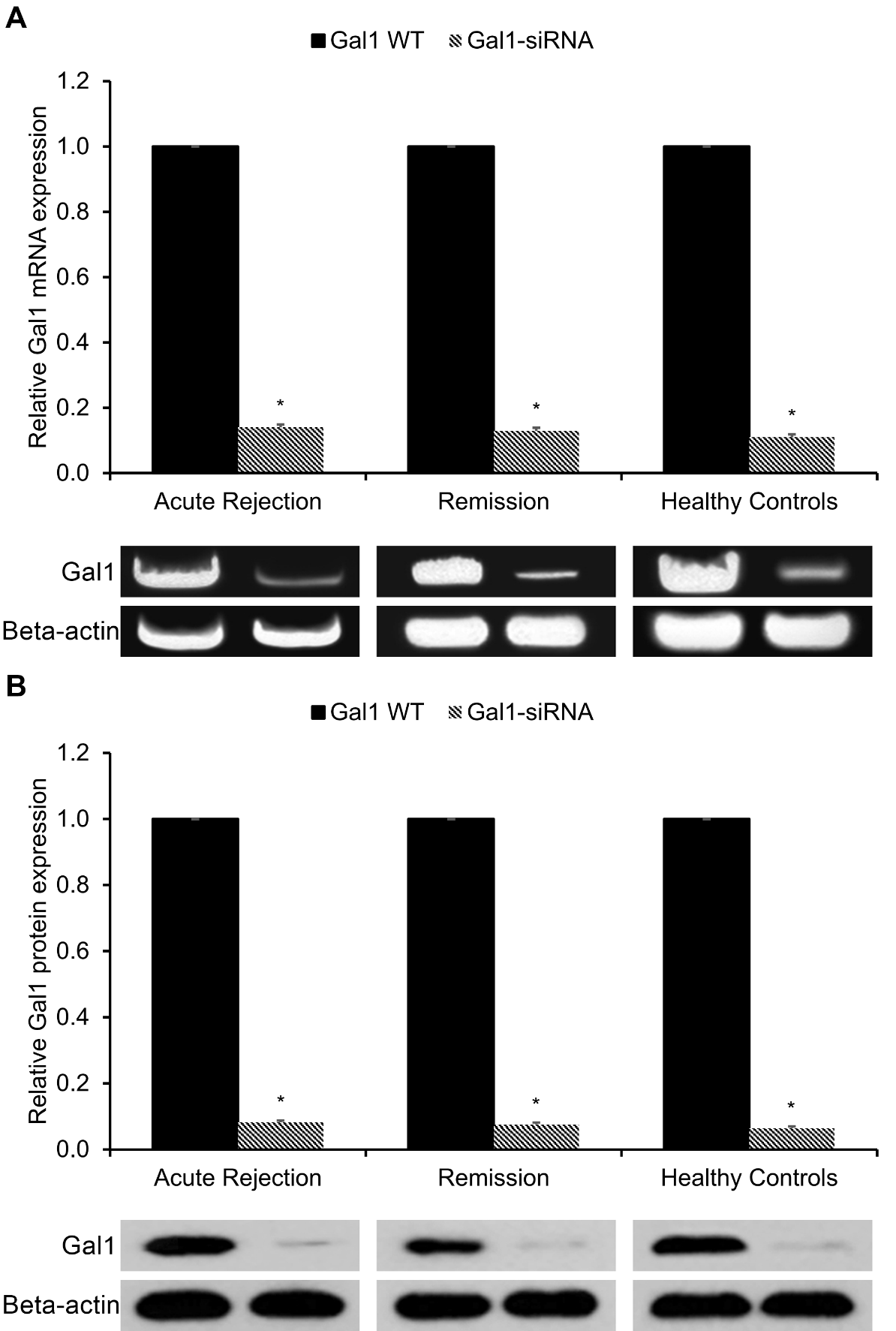
**
