# Supplementary material for: Dysfunctional immunoregulation in human liver allograft rejection associated with compromised galectin-1/CD7 pathway function
Source: Cell Death Dis. 2018 Feb 20;9(3):293. doi: 10.1038/s41419-017-0220-3 (PMC5833641; doi:10.1038/s41419-017-0220-3)

**Supplementary Figure 3. Responder T-Cell CD43 and CD45 Expression Positively Correlates with CD7 Expression**

(A) Representative dot plots of CD43 PE (*x*-axis) versus CD25 PE-Cy7 (*y*-axis) from a healthy control participant (left panel), a transplant patient in remission (middle panel), and an acute rejection transplant patient (right panel). Cells were gated on CD4+ T-cells. (B) Percentage of CD43+CD25- cells in healthy controls (n = 40), transplant patients in remission (n = 85), and acute rejection transplant patients (n = 31). (C) Statistically significant positive correlation between CD43 and CD7 expression on CD4+CD25- responder T-cells. (D) Representative dot plots of CD45 PE (*x*-axis) versus CD25 PE-Cy7 (*y*-axis) from a healthy control participant (left panel), a transplant patient in remission (middle panel), and an acute rejection transplant patient (right panel). Cells were gated on CD4+ T-cells. (E) Percentage of CD45+CD25- cells in healthy controls (n = 40), transplant patients in remission (n = 85), and acute rejection transplant patients (n = 31). (F) Statistically significant positive correlation between CD45 and CD7 expression on CD4+CD25- responder T-cells. Each experiment was performed in triplicate. Results are reported as means ± standard errors of the mean (SEMs). **P*<0.05 versus healthy controls group, †*P*<0.05 versus remission group.


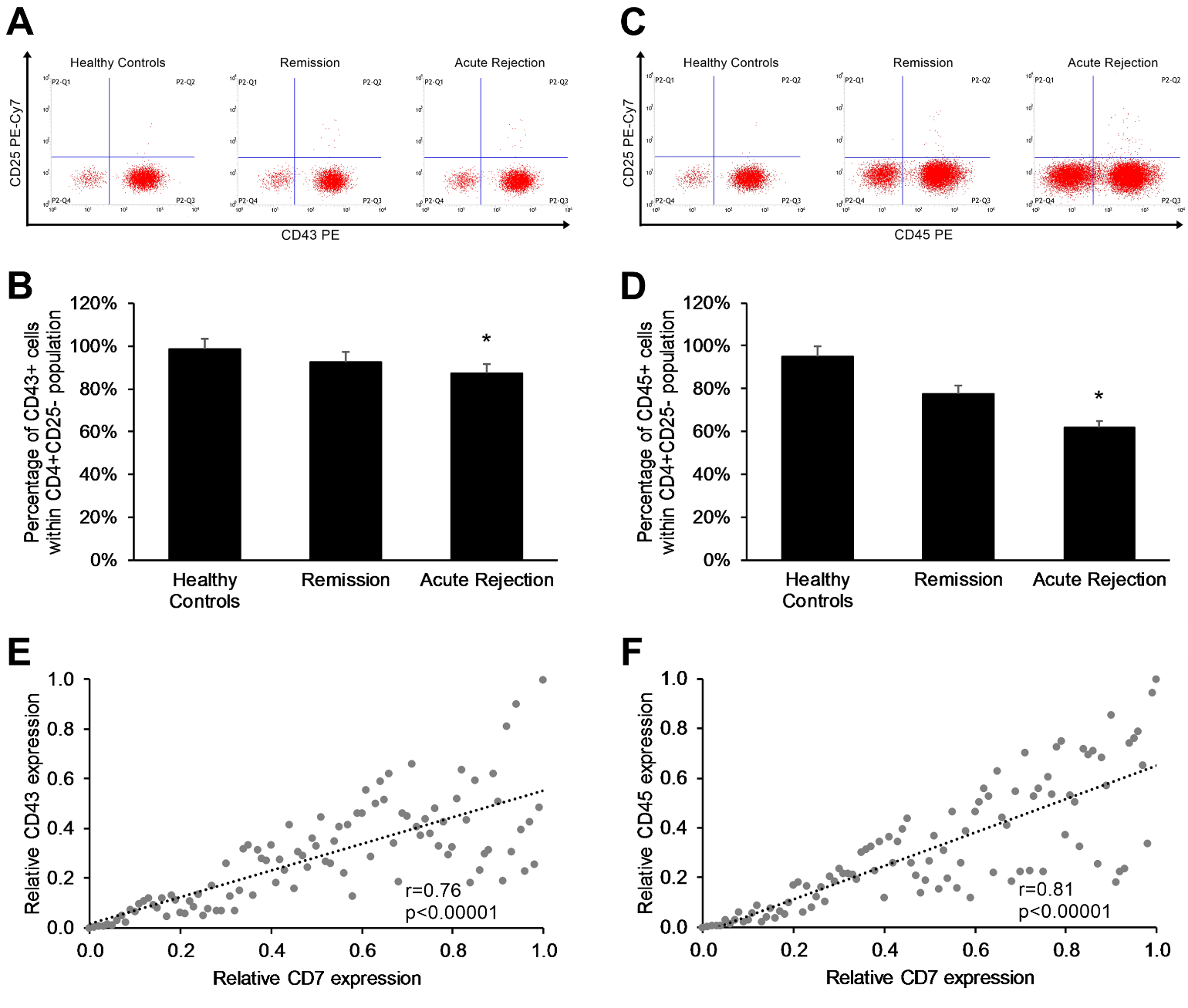

Supplement: Supplementary file 3 — Supplementary Figure 3 [file 41419_2017_220_MOESM3_ESM.docx]
