# Supplementary material for: Dysfunctional immunoregulation in human liver allograft rejection associated with compromised galectin-1/CD7 pathway function
Source: Cell Death Dis. 2018 Feb 20;9(3):293. doi: 10.1038/s41419-017-0220-3 (PMC5833641; doi:10.1038/s41419-017-0220-3)

**Supplementary Figure 4. Responder T-Cell CD43 Expression Positively Correlates with T-Reg Suppression and Promotes T-Reg Suppression in a Gal1-Dependent Manner**

Unfractionated regulatory T-cells (CD4+CD25+ cells) isolated from acute rejection transplant patients (n = 31), transplant patients in remission (n = 85), and healthy control subjects (n = 40) were added to (A) CD7+CD43_low_ or CD7+CD43_high_ responder T-cells or (C) CD7+CD45_low_ or CD7+CD45_high_ responder T-cells. After a five-day co-culturing period, ^3^H-thymidine incorporation was applied to measure % suppression of responder T-cell proliferation (a measure of regulatory T-cell suppressor function). (B) Statistically significant positive correlation between responder T-cell CD43 expression and % suppression of responder T-cell proliferation. (D) However, there was no statistically significant correlation between responder T-cell CD45 expression and % suppression of responder T-cell proliferation. (E) Gal1 WT and Gal1-silenced regulatory T-cells (CD4+CD25+ cells) isolated from acute rejection transplant patients (n = 31), transplant patients in remission (n = 85), and healthy control subjects (n = 40) were added to CD7+CD43_low_ or CD7+CD43_high_ responder T-cells. ^3^H-thymidine incorporation was applied to measure % suppression of responder T-cell proliferation as described above. Each experiment was performed in triplicate. Results are reported as means ± standard errors of the mean (SEMs). **P*<0.05 as indicated. †*P*<0.05 versus matching Gal1 WT group.


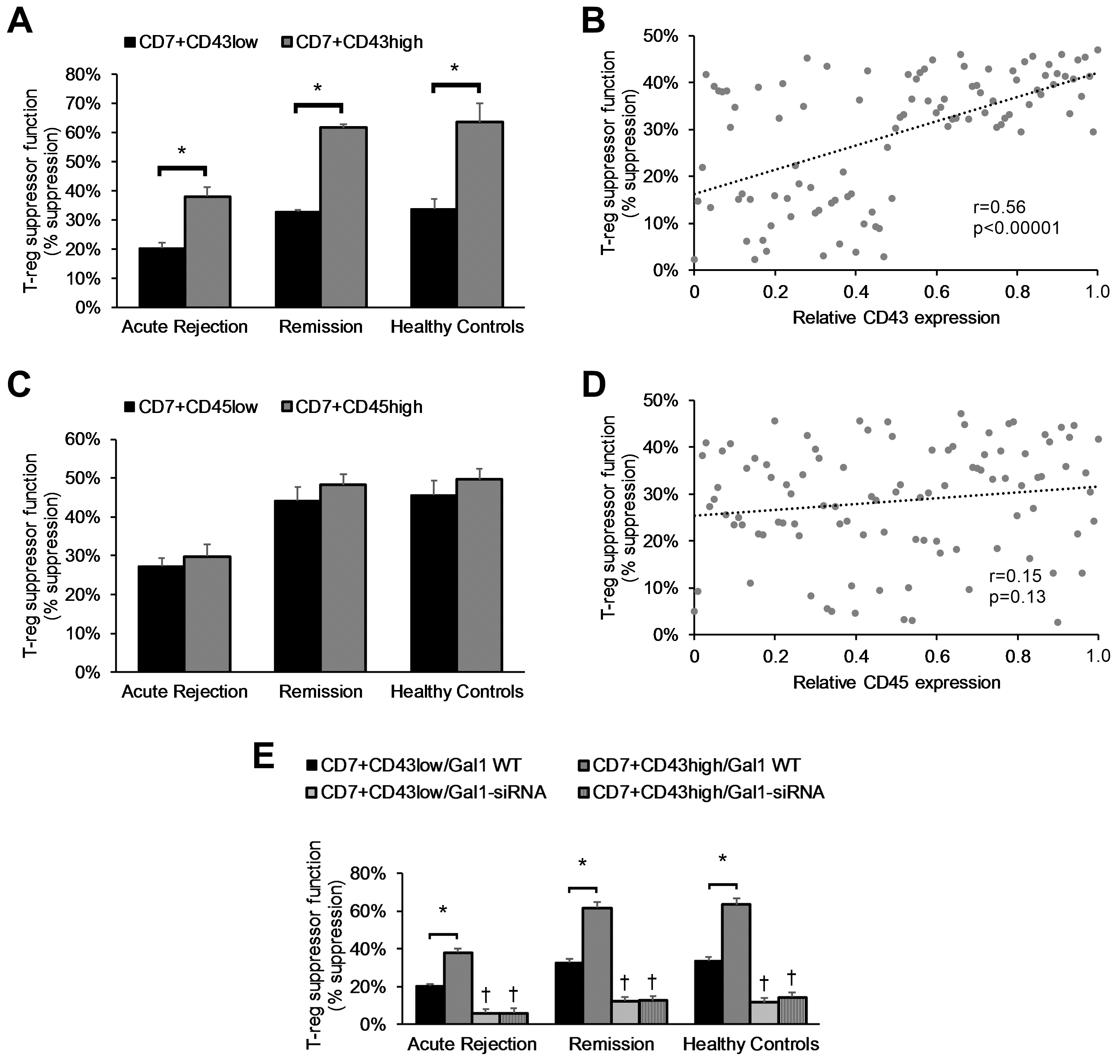

Supplement: Supplementary file 4 — Supplementary Figure 4 [file 41419_2017_220_MOESM4_ESM.docx]
