# Supplementary material for: Dysfunctional immunoregulation in human liver allograft rejection associated with compromised galectin-1/CD7 pathway function
Source: Cell Death Dis. 2018 Feb 20;9(3):293. doi: 10.1038/s41419-017-0220-3 (PMC5833641; doi:10.1038/s41419-017-0220-3)
Supplement: Supplementary file 5 — Supplementary Table 1 [file 41419_2017_220_MOESM5_ESM.docx]

**Supplementary Table 1. Clinical Correlation Analysis**

| **Biomarker** | **Clinical Correlate** | ***r*** |
| --- | --- | --- |
| CD7+_all_ | AST | -0.46 |
| CD7+_all_ | ALT | -0.45 |
| CD7+_all_ | GGT | -0.47 |
| CD7+CD43_high_ | AST | -0.61 |
| CD7+CD43_high_ | ALT | -0.59 |
| CD7+CD43_high_ | GGT | -0.62 |
| CD7+CD45_high_ | AST | -0.86 |
| CD7+CD45_high_ | ALT | -0.84 |
| CD7+CD45_high_ | GGT | -0.89 |
| CD7- T-bet+ | AST | 0.67 |
| CD7- T-bet+ | ALT | 0.66 |
| CD7- T-bet+ | GGT | 0.69 |
