# Supplementary material for: Dysfunctional immunoregulation in human liver allograft rejection associated with compromised galectin-1/CD7 pathway function
Source: Cell Death Dis. 2018 Feb 20;9(3):293. doi: 10.1038/s41419-017-0220-3 (PMC5833641; doi:10.1038/s41419-017-0220-3)
Supplement: Supplementary file 6 — Summary of Supplementary Information [file 41419_2017_220_MOESM6_ESM.docx]

**SUPPLEMENTARY INFORMATION**

**Supplementary Figure 1. Effect of IL-10 on Regulatory T-Cell Suppression of Responder T-Cells**

**Supplementary Figure 2. Validation of Gal1 Knockdown by Gal1 Silencing RNA (Gal1-siRNA)**

**Supplementary Figure 3. Responder T-Cell CD43 and CD45 Expression Positively Correlates with CD7 Expression**

**Supplementary Figure 4. Responder T-Cell CD43 Expression Positively Correlates with T-Reg Suppression and Promotes T-Reg Suppression in a Gal1-Dependent Manner**

**Supplementary Table 1. Clinical Correlation**
